# Key Contributors to Signal Generation in Frequency Mixing Magnetic Detection (FMMD): An In Silico Study

**DOI:** 10.3390/s24061945

**Published:** 2024-03-18

**Authors:** Ulrich M. Engelmann, Beril Simsek, Ahmed Shalaby, Hans-Joachim Krause

**Affiliations:** 1 Medical Engineering and Applied Mathematics, FH Aachen University of Applied Sciences, 52428 Jülich, Germany; 2 Institute of Biological Information Processing, Bioelectronics (IBI-3), Forschungszentrum Jülich, 52428 Jülich, Germany; 3 Institute of Nano- and Biotechnologies (INB), FH Aachen University of Applied Sciences, 52428 Jülich, Germany

**Keywords:** magnetic nanoparticles, micromagnetic simulation, magnetic relaxation, frequency mixing magnetic detection, coupled Néel–Brownian relaxation dynamics, magnetic biosensing, key performance indicators

## Abstract

Frequency mixing magnetic detection (FMMD) is a sensitive and selective technique to detect magnetic nanoparticles (MNPs) serving as probes for binding biological targets. Its principle relies on the nonlinear magnetic relaxation dynamics of a particle ensemble interacting with a dual frequency external magnetic field. In order to increase its sensitivity, lower its limit of detection and overall improve its applicability in biosensing, matching combinations of external field parameters and internal particle properties are being sought to advance FMMD. In this study, we systematically probe the aforementioned interaction with coupled Néel–Brownian dynamic relaxation simulations to examine how key MNP properties as well as applied field parameters affect the frequency mixing signal generation. It is found that the core size of MNPs dominates their nonlinear magnetic response, with the strongest contributions from the largest particles. The drive field amplitude dominates the shape of the field-dependent response, whereas effective anisotropy and hydrodynamic size of the particles only weakly influence the signal generation in FMMD. For tailoring the MNP properties and parameters of the setup towards optimal FMMD signal generation, our findings suggest choosing large particles of core sizes $d_{C}>25$ nm with narrow size distributions (σ<0.1) to minimize the required drive field amplitude. This allows potential improvements of FMMD as a stand-alone application, as well as advances in magnetic particle imaging, hyperthermia and magnetic immunoassays.

## 1. Introduction

Magnetic nanoparticles (MNPs) attract wide interest in innovative biomedical application [1,2]. They are applied as diagnostic imaging tracers in magnetic particle imaging (MPI) [3,4], therapeutic heating agents in magnetic fluid hyperthermia (MFH) [5,6] and flexible sensors in magnetic biosensing [7,8]. While MPI and MFH have been relatively widespread in global research for the past 15 years, the area of magnetic biosensing using MNPs is still being established with novel methods being introduced. Among those promising methods, magnetic frequency mixing detection (FMMD) is becoming increasingly popular, with potential to combine analyte-differentiating biosensing techniques [9], even with MPI imaging modalities simultaneously [10]. To achieve this, FMMD uses a dual frequency excitation to drive MNPs through saturation and generate a nonlinear magnetic response from the particles [11], which is characterized by a multi-faceted intermodulation signal that allows for multiplex interpretation and consequently high information gain per measurement [12]. Due to the small nano-sized tracers and high sensitivity, FMMD is especially suitable for detection of structures on the micro- and nanoscale, as shown by its successful application for detection of SARS [13] and influenza viruses [14,15], antibodies [16] and aflatoxin B1 [17] as well as nanoparticle size differentiation [18].

All of the above techniques are based on the magnetic particle dynamic relaxation in an alternating magnetic field (AFM), either for direct imaging (MPI), direct heat generation (MFH) or indirectly measured changes in the relaxation state of MNP (FMMD). As such, the dynamic relaxation process of MNP has been intensively studied theoretically and via simulations in the past, with successes for both MPI and MFH applications [19,20,21]. However, such comprehensive simulation-based studies have, to the best of the authors’ knowledge, not yet been carried out for FMMD. Therefore, it is currently unknown what key contributors rule the FMMD signal overall and how exactly the AFM parameters (setup) could be matched to the intrinsic particle properties (MNP) to generate an optimum FMMD signal. Knowledge of such key contributing parameters and the consequent optimal matching of setup to MNP parameters could provide leverage to position FMMD besides established biomedical applications such as MPI and MFH, guide synthesis of optimized MNP [22,23] or introduce FMMD as an adjunct to these for a theranostic platform, as has been demonstrated for MPI–MFH combinations [24,25,26]. In the following, we use coupled Néel–Brownian stochastic magnetic relaxation dynamics simulations to close this gap, investigating the optimal signal generation in FMMD. The simulation framework has been successfully applied to MPI [27] and MFH [28], been extended to FMMD and compared to Langevin theory [29], as well as experimentally validated [30]. Such an in silico study allows to assess the individual and isolated influence of key contributions to signal generation over a wide range. The key contributors varied here are particle core size (i.e., diameter), the hydrodynamic size, the effective magnetic anisotropy and the core-size distribution width, as well as the frequency and field amplitude.

## 2. Materials and Methods

### 2.1. Magnetic Relaxation Theory

The applied alternating magnetic field (AMF) is described by

(1) $$H\left(t\right)=H_{0}+H_{1}sin\left(2\pi f_{1}t\right)+H_{2}sin\left(2\pi f_{2}t\right)\;,$$

where $H_{0}$ denotes the static magnetic offset field, $H_{1}$ is the excitation field amplitude at high frequency $f_{1}$ and the drive field amplitude $H_{2}$ at low frequency $f_{2}$.

The particle relaxation dynamics at such an applied AMF, H, can be described by combined Néel–Brownian relaxation [31]. The Néel relaxation of a single MNP core, $m_{p}$, is given by the Landau–Lifshitz–Gilbert equation (LLG) [32]:

(2) $$\frac{dm_{p}}{dt}=\frac{\mu_{0}\gamma }{1+\alpha^{2}}\cdot \left(H_{eff}\times m_{p}+\alpha m_{p}\times \left(H_{eff}\times m_{p}\right)\right)$$

with the permeability of free space, $\mu_{0}$, the electron gyromagnetic ratio, γ, the damping parameter, α, and the effective field $H_{eff}$. The Brownian rotation of a single particles easy axis, n, can be described by a generalized torque (GT), Θ, as follows [33]:

(3) $$\frac{dn}{dt}=\frac{\Theta }{6\eta V_{H}}\times n$$

with the carrier matrix viscosity, η, and the MNP hydrodynamic volume, $V_{H}=\frac{\pi }{6}\cdot d_{h}^{3}$, in which $d_{H}$ is the hydrodynamic particle size. Néel and Brownian relaxations are both coupled using particle internal energy:

(4) $$U=-\mu_{0}\cdot m_{p}\left(m_{p}\cdot H\right)-K_{eff}{\cdot V}_{c}{\left(m_{p}\cdot n\right)}^{2}$$

where $m_{p}=\left|m_{p}\right|=V_{c}\cdot M_{S}$ gives the magnitude of the MNP magnetic moment, and $V_{c}=\frac{\pi }{6}\cdot d_{c}^{3}$ is the MNP core volume. The first term in Equation (4) represents the Zeeman energy with the applied AMF, H, while the second term represents the magnetic anisotropy energy, $K_{eff}\cdot V_{c}$, and uses the assumption of uniaxial anisotropy and spherically shaped particles, approximated by an effective anisotropy constant.

Thermal fluctuations are taken into account by expanding the LLG and GT (Equations (1) and (2)) with $H_{\mathrm{th}}$ and $\Theta_{th}$, which add Gaussian-distributed white noise with zero mean as follows: $\langle H_{th}^{i}\left(t\right)\rangle =0$ and $\langle \Theta_{th}^{i}\left(t\right)\rangle =0$ and variances, respectively: $\langle H_{th}^{i}\left(t\right)H_{th}^{j}\left(t^{\prime }\right)\rangle =\frac{2k_{B}\;T\cdot \left(1+\alpha^{2}\right)}{\gamma m_{p}\alpha {\cdot \delta }_{ij}\delta \left(t-t^{\prime }\right)}$ and $\langle \Theta_{th}^{i}\left(t\right)\Theta_{th}^{j}\left(t^{\prime }\right)\rangle =12k_{B}T\eta V_{H}\cdot \delta_{ij}\delta \left(t-t^{\prime }\right)$, where T represents the global temperature of the entire ensemble. Implementing these fluctuations changes the effective field and generalized torque as follows:

(5) $$H_{eff}=-\frac{1}{m_{p}\cdot \mu_{0}}\cdot \frac{\partial U}{\partial m}+H_{th}=H+\frac{2K_{eff}\cdot V_{c}}{m_{p}\cdot \mu_{0}}\cdot \left(m_{p}\cdot n\right)n+H_{th}$$

(6) $$\Theta =\frac{\partial U}{\partial n}\times n+\Theta_{th}=-2K_{eff}\cdot V_{c}\left(m_{p}\cdot n\right)\left(m_{p}\times n\right)+\Theta_{th}$$

### 2.2. Simulation Implementation & Framework

To solve the system of coupled stochastic differential formed by Equations (2)–(6), the Stratonovich–Heun scheme is applied. Further details on the step-by-step implementation are found in previous publications [31,34]. The general open-access Python source code is available as referenced in the Data Availability Statement.

The damping parameter α was set to unity [35]. A total of 1000 particles formed a simulation ensemble. Each particle was initialized with randomized direction of magnetization and easy axes. The MNP were then thermalized for 1/5 of the total number of time steps, N, before the AMF was applied. The number of time steps was set to N=75,000, equaling a time step size of approx. 10 ns. Each individually simulated ensemble was allowed to evolve under the AFM for the duration of two full cycles of the (lower) drive frequency, *f*_2_, to capture the dynamic magnetization effects. The final magnetization of one simulation run was averaged over a series of five independently simulated ensembles to achieve suitable compromise between statistical accuracy and required computation time. Calculations were carried out on a PC cluster consisting of 2 × Intel Xeon 8168 CPUs with 2.7/3.7 GHz and 24 clusters each and 768 GB RAM at 2666 MHz.

### 2.3. Simulation Input: Key Parameters Varied

The above mathematical description of the magnetic relaxation process of MNP (Equations (2)–(6)) directly allows to identify the key contributors to the FMMD signal as follows:

1. The intrinsic physical properties of the MNP: the particle core size, $d_{C}$, its size distribution width, $\sigma_{d_{C}}$, the effective anisotropy constant, $K_{eff}$, as well as the hydrodynamic size, $d_{H}$.
2. The external influences the applied field parameters of the AFM: the excitation frequency, $f_{1}$, and the drive field amplitude, $H_{2}$. Note that the drive frequency, $f_{2}=2$ kHz, and excitation field amplitude, $H_{1}=1.2$ mT/µ_0_, are expected to contribute much less to the FMMD signal generation as they are at least one order of magnitude lower than their respective counterparts [36]; therefore, they are fixed for all simulations.

We varied the above-mentioned parameters over the ranges listed in Table 1, reproducing experimentally reasonable ranges for each specific parameter: e.g., core sizes between $d_{C}=10$ nm and 30 nm with hydrodynamic sizes of up to 200 nm are easily and reproducibly synthesized [37,38] and of main interest for more established medical applications of MPI and MFH [38,39,40]. The effective anisotropy values were varied widely below (e.g., 5 kJ/m^3^ [41]) and above (e.g., up to 20 kJ/m^3^ [42]) bulk value ($K_{eff,{Fe}_{3}O_{4}}=-11$ kJ/m^3^ [43]), as the exact value for nanostructured magnetite is still of ongoing discussion (see Discussion Section 4.1 for details). The field parameters for $H_{2}$ and $f_{1}$ were chosen to match the typical values of the experimental FMMD setups [44]. For each key contributing parameter not varied in a specific simulation run, we fixed the values to standard values commonly used in our labs, i.e., MNP sizes of $d_{C}=20$ nm and $d_{H}=130$ nm, (representing Perimag^®^ available from Micromod Partikeltechnologie GmbH, Rostock, Germany). For effective anisotropy, we chose the bulk value for magnetite. The size distribution width, however, was chosen purposefully very low (but still realistic from synthesis [45]), $\sigma_{d_{c}}=0.05$, to suppress overlapping effects of particle core size or, in other words, to allow a mostly isolated key parameter variation. The reason is that the nonlinear response of the largest particles is expected to strongly dominate the measured signal [24,28]; therefore, a wide size distribution would obscure the response of small particles. Field parameters with $H_{2}=16$ mT/µ_0_ and $f_{1}=40$ kHz were based on experimental performance of our custom-build FMMD setup [44]. The following parameters are not varied and fixed at the following values: the saturation magnetization for magnetite, $M_{S}=476$ kA/m [36], the viscosity of the water, η=0.89 mPa·s, and the operating temperature, T=310 K (a typical value for the temperature inside the measurement head due to resistive heating from the field coils) [36].

If a parameter is not varied during simulation, it is kept at the following representative value: core size $d_{C}=20$ nm, core-size distribution width $\sigma_{d_{C}}=0.05$, hydrodynamic size $d_{H}=130$ nm, effective anisotropy $K_{eff}=-11$ kJ/m^3^, drive field amplitude $H_{2}=$16 mT/µ_0_ and excitation frequency $f_{1}=40$ kHz, while excitation field amplitude $H_{1}=1.2$ mT/µ0 and drive frequency $f_{2}=2$ kHz are always kept constant (see Table 1).

Note that special attention was given to the variation of the effective anisotropy constant, $K_{eff}$, which was simulated for an additional value of $d_{C}=28$ nm besides the standard value of $d_{C}=20$ nm and core-size distribution width $\sigma_{d_{C}}=0.05$ and 0.3 (see also Table 1). These values were chosen to probe maximum impact of $K_{eff}$-variation on the FMMD signal. The rationale for this is given by the size-dependency of anisotropy, which is further elaborated and discussed in Section 4.1

## 3. Results

In the following, we present the individual effects that each parameter variation has on the FMMD signal intensities of the components at the mixing frequencies $f_{1}+nf_{2}\;\left(n=1,\;2,\;3,\;4\right)$ as a function of the static magnetic offset field in the range of *H*_0_ = (0, 1, …, 24) mT/*µ*_0_. For convenient comparison, the (arbitrary) FMMD signal intensities (*y*-axis) are equally scaled for each intermodulation signal ($f_{1}+nf_{2})$, using the same scaling for all the key parameters being varied.

### 3.1. Dependency on Intrinsic Particle Properties: $d_{C},\sigma_{d_{C}},K_{eff},d_{H}$

**Figure 1:**
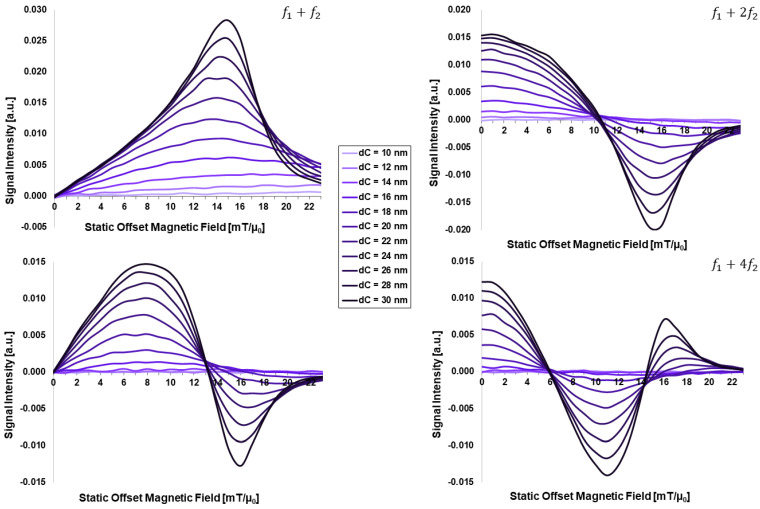
Core-size ($d_{C}$)-dependent FMMD signal intensity for mixing frequencies $f_{1}+n\cdot f_{2}$ with n=1, 2, 3, 4. All input parameters are set according to Table 1.

Figure 1 presents the core-size-dependent FMMD signal intensity as a function of the static magnetic offset field *H*_0_ for particles of different core diameters in the range of $d_{C}=(10,\;\ldots ,\;30$) nm. All four simulated frequency mixing components $f_{1}+nf_{2}$ show a steadily increasing signal intensity with increasing core size, in accordance with previous works [30].

Particles below $d_{C}=16$ nm generate (almost) no signal, while a distinct peak is noticeable in the mixing component $f_{1}+f_{2}$ for $d_{C}=18$ nm, which becomes gradually narrower and more pronounced for larger core sizes. The peak is asymmetrical with a steep right-hand shoulder, which is becoming steeper with increasing core size.

**Figure 2:**
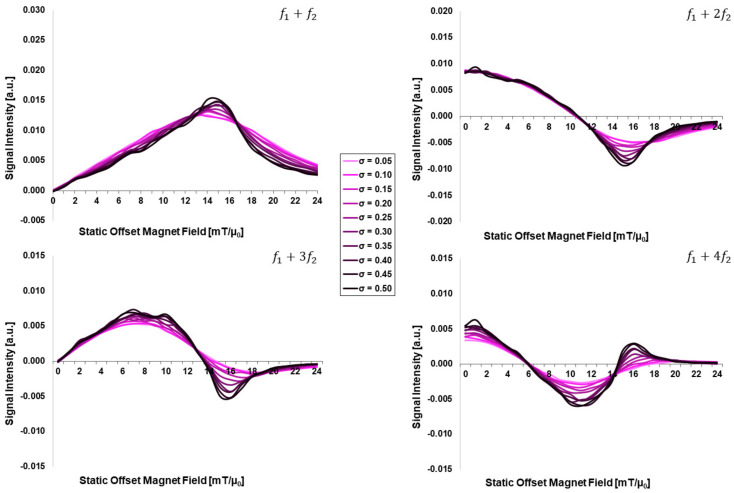
Core-size distribution width (${\sigma_{d}}_{C}$)-dependent FMMD signal intensity for mixing frequencies $f_{1}+n\cdot f_{2}$ with n=1, 2, 3, 4. All input parameters are set according to Table 1.

Figure 2 presents the core-size distribution width-dependent FMMD signal intensities as a function of the static magnetic offset field *H*_0_ for different core-size distribution widths $\sigma_{d_{C}}=(0.05,\;\ldots ,\;0.50$) for a mean core size of $d_{C}=20$ nm. Three effects are observed, steadily increasing for increasing distribution width *σ*:

First, the peak intensities of the mixing terms $f_{1}+f_{2}$ and $f_{1}+{2f}_{2}$ increase slightly.

Second, the peak width decreases slightly, especially for higher values of static offset field, $H_{0}$, beyond the peak position. This leads to an increasingly asymmetric signal peak, with a steeper right flank.

And third, the signal intensities overall show more fluctuations, i.e., a less smooth signal profile. All these effects are attributed to the influence of larger-than-mean core sizes $d_{C}>20$ nm that dominate the signal intensity (see Section 4.1 for detailed discussion).

**Figure 3:**
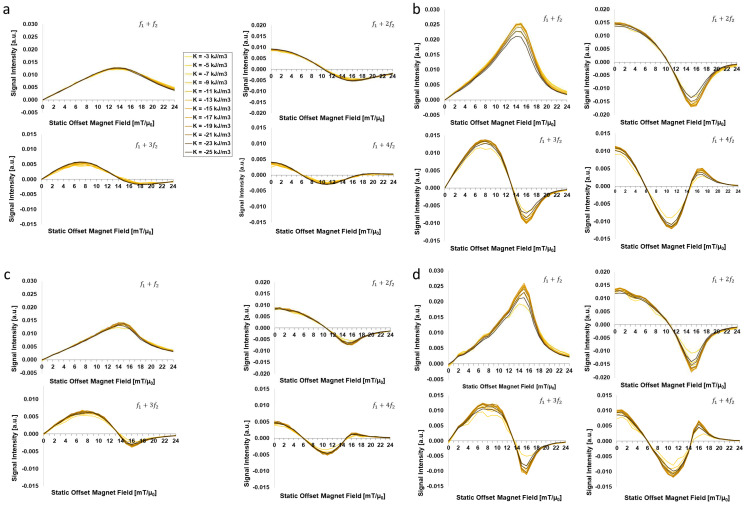
Effective anisotropy constant ($K_{eff}$)-dependent FMMD signal intensity for mixing frequencies $f_{1}+n\cdot f_{2}$ with n=1, 2, 3, 4 for the core-size distributions of (**a**) $d_{C}=20$ nm and $\sigma_{d_{C}}=0.05$ (standard values), (**b**) $d_{C}=28$ nm and $\sigma_{d_{C}}=0.05$, (**c**) $d_{C}=20$ nm and $\sigma_{d_{C}}=0.3$ and (**d**) $d_{C}=28$ nm and $\sigma_{d_{C}}=0.3.$ All other input parameters are set according to Table 1.

Figure 3 presents the FMMD signal intensity as a function of the static magnetic offset field *H*_0_ for different effective anisotropy constants *K*_eff_ = (−3, −5, …, −25) kJ/m^3^ and for certain combinations of core-size distribution parameters, including $d_{C}=\left(20,\;28\right)\;nm$ and $\sigma_{d_{C}}=\left(0.05,\;0.3\right)$. It demonstrates a slight decrease in FMMD signal peak intensity for low $K_{eff}$-values for mid-size particles, $d_{C}=20$ nm. This dependency is more pronounced for narrow size distributions, $\sigma_{d_{C}}=0.05$ (Figure 3a, up to −9 kJ/m^3^) than for wide ones, $\sigma_{d_{C}}=0.3$ (Figure 3c, up to −5 kJ/m^3^).

The Impact of effective anisotropy on FMMD signal generation is more prominent for large particles, $d_{C}=28$ nm, which generate up toapprox. 50% higher peak signal intensities in direct comparison to $d_{C}=20$ nm. For these larger particles, a distinct decrease of up toapprox. 25% is observed for wider size distributions, $\sigma_{d_{C}}=0.3$ for $K_{eff}$-values below −7 kJ/m^3^ as well as above −19 kJ/m^3^. (Figure 3d). The same trend is equally noticeable but slightly less strong (up to approx. 20%) for narrow size distributions, $\sigma_{d_{C}}=0.05$ (Figure 3b). This observation is further elaborated and discussed in Section 4.1.

**Figure 4:**
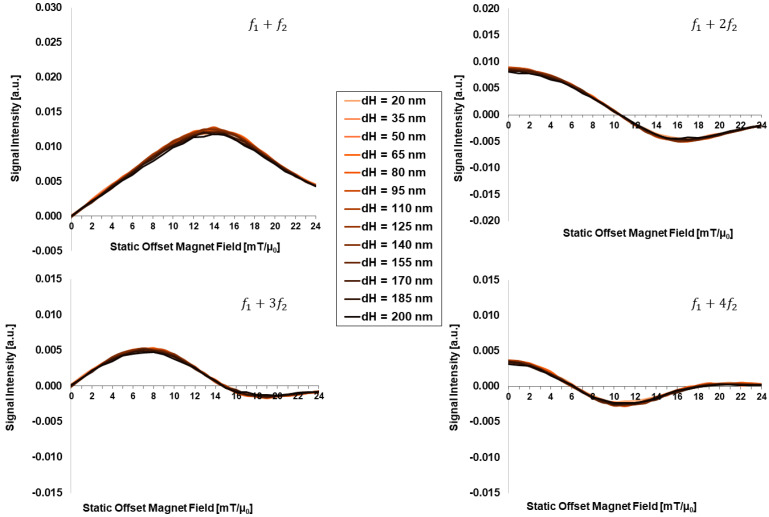
Hydrodynamic-size ($d_{H}$)-dependent FMMD signal intensity for mixing frequencies $f_{1}+n\cdot f_{2}$ with n=1, 2, 3, 4. All other parameters are set according to Table 1.

Figure 4 presents the offset-field-dependent FMMD signal intensity for different hydrodynamic size of the particles in the range of $d_{H}=(20,\;\ldots ,\;200$) nm. Across this range of $d_{H}$-values, there is no remarkable change detected in the FMMD signal intensity profiles of all four intermodulation signals ($f_{1}+nf_{2})$. Thus, the FMMD signal generation is considered independent of the hydrodynamic size in this range.

### 3.2. Dependency on External Applied Field Parameters: $H_{2},f_{1}$

**Figure 5:**
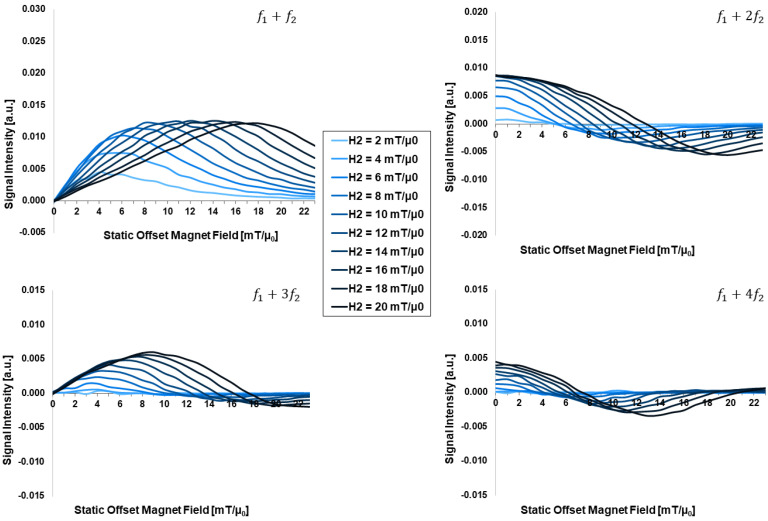
Drive-field-dependent ($H_{2}$) FMMD signal intensity for mixing frequencies $f_{1}+n\cdot f_{2}$ with n=1, 2, 3, 4. All input parameters are set according to Table 1.

Figure 5 presents the drive-offset-field-dependent FMMD signal intensity for different drive field amplitudes in the range of $H_{2}=\left(2,\;\ldots ,\;20\right)$ mT/µ_0_. Two general observations are made:

First, the FMMD signal peak intensity increases between $H_{2}=\left(2,\;\ldots ,\;10\right)$ mT/µ_0_ and remains steady for $H_{2}>10$ mT/µ_0_ in case of the $f_{1}+f_{2}$ component, and increases slightly across the entire range of $H_{2}$-values for the other three intermodulation signals with $f_{1}+nf_{2},\;n>1$, respectively.

Second, with increasing $H_{2}$-value, the positions of the (local) intensity extrema and of the zero crossing(s) of mixing terms ($f_{1}+nf_{2},\;n>1$) both shift continuously towards larger offset fields, $H_{0}$. For further discussion, see Section 4.2 below.

**Figure 6:**
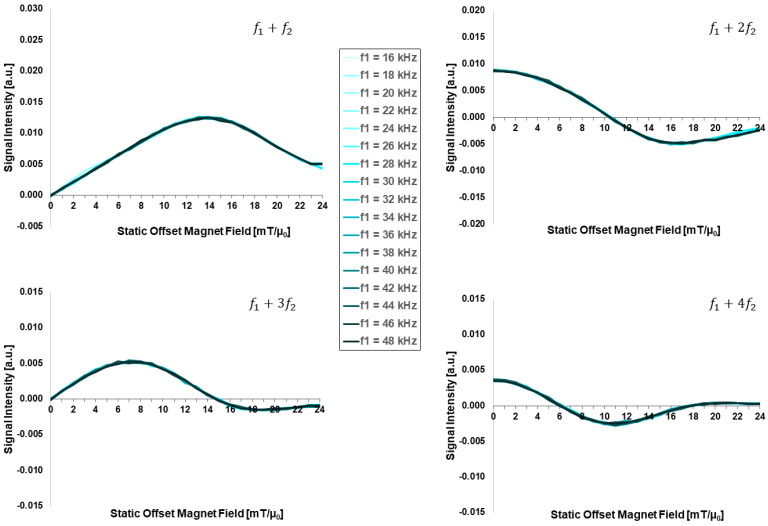
High-frequency ($f_{1}$)-dependent FMMD signal intensity for mixing frequencies $f_{1}+n\cdot f_{2}$ with n=1, 2, 3, 4. All input parameters are set according to Table 1.

Figure 6 presents the excitation-field high-frequency-dependent FMMD signal intensity in the range of $f_{1}=\left(16,\;\ldots ,\;48\right)$ kHz. Across this range of excitation frequency values, there is no remarkable change detected in the FMMD signal intensity profiles of all four intermodulation signals ($f_{1}+nf_{2})$. Thus, the FMMD signal generation is considered independent of the excitation frequency in this range.

### 3.3. Summary of Effects of Key Parameters

The effects on the FMMD signal generation in dependence of the isolated key parameter variation are listed in Table 2 below. It compares the influence on peak signal intensity value, the peak width and the shape of the intensity profile across all key parameters varied qualitatively.

## 4. Discussion

Overall, the results of isolated parameter variation of the key contributing parameters delineate the dominating effect of MNP core size ($d_{C}$) to FMMD signal generation (see Table 2). The results will be discussed in detail, following the organization of Section 3: In Section 4.1, we study them from the perspective of the intrinsic physical properties of MNP, and in Section 4.2, we examine the dependence on the external field parameters, and in Section 4.3, limitations and possible improvements of our simulation method are given.

### 4.1. FMMD Dependency on Intrinsic Particle Properties: $d_{C},\sigma_{d_{C}},K_{eff},d_{H}$

The strong and dominating dependency of FMMD signal generation on the MNP core size (Figure 1) is in accordance with the results of the well-established single-frequency excitation technique of MPI [46,47,48] and MFH [28,49,50]: all these studies generally agree on an optimal MNP core size for relaxation-dependent applications with $f\sim \left(10-100\right)$ kHz to be in the twenties of nanometers, around ~25 nm (the exact value may vary due to the specific MNP properties and excitation setup characteristics).

In stark contrast, the hydrodynamic size showed no impact on the FMMD signal generation (compare to Figure 4), even though it is considered in the GT expression directly at the power of three (compare to Equations (3) and (4)). However, Brownian relaxation (GT) processes have been argued to be mostly relevant for (a) non-interacting, monodisperse MNP above $d_{C}=20$ nm at frequencies of f~1 kHz [51] or (b) large particles, especially agglomerates of sizes above several hundreds of nanometers at frequencies of f~100 kHz [52,53]. Therefore, as for all simulations $f_{1}\gg 1$ kHz and non-interacting, (nearly) monodisperse ($\sigma_{d_{C}}=0.05$) particle distributions hold, argumentation (a) confirms the independence of $d_{H}$ for FMMD signal intensity generation. For future studies, a more complex address of the $d_{H}$-dependence by including agglomeration and/or interaction effects will be discussed as limitations in Section 4.3.

However, all studies mentioned above also address the complex nature of the interplay of (core) size distribution and magnetic anisotropy for predicting the ideal signal generation constituents for any given situation. This will therefore be elaborated further in the following.

As an increasing core size, $d_{C}$, distinctively increases the FMMD signal intensity peak (Figure 1), the slightly increased signal intensity peak for increasing core-size distribution width, $\sigma_{d_{C}}$, (Figure 2 for $d_{C}=20$ nm) can be explained as follows: as $\sigma_{d_{C}}$ increases, larger as well as smaller particle sizes are introduced in the ensemble of MNP. While the smaller particles ($d_{C}\ll 20$ nm) are not adding signal contribution (see Figure 1), the larger particles above $d_{C}=20$ are dominating the signal. This causes both the increase in peak intensity as well as the gradual narrowing of the peak width; however, this effect is less pronounced than in the isolated core-size variation (Figure 1), since the larger particles are not numerous (note that the ensemble is limited to 1000 particles per simulation run, Section 2.2). This knowledge is of practical relevance, since we were able to predict the core-size distribution of a real system of MNP from experimentally measured FMMD signals using the presented simulation framework recently [30]. However, the nature of magnetic anisotropy contributions remains unknown until now. Yet, the present work with its isolated parameter variations allows us to take these contributions into account systematically as follows.

**Figure 7:**
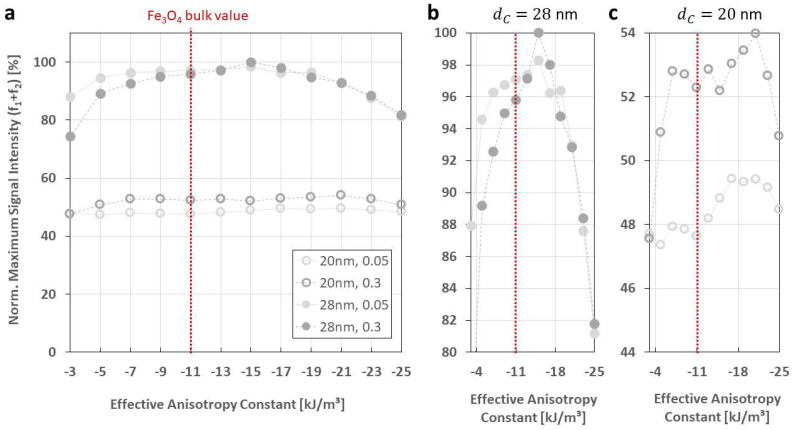
Comparison of maximum (peak) signal intensity values for the first intermodulation signal, $f_{1}+f_{2}$, with respect to the effective anisotropy constant. (**a**) given for $d_{C}=20$ nm (open symbols) and 28 nm (solid symbols) with $\sigma_{d_{C}}=0.05$ (solid light line) and $\sigma_{d_{C}}=0.3$ (dashed dark line) and (**b**,**c**) showing a zoom for 28 nm and 20 nm, respectively. The magnetite bulk value is marked as a red dotted line at $K_{eff,{Fe}_{3}O_{4}}^{bulk}=-11$ kJ/m^3^. Values are extracted from Figure 3 and normalized to the highest signal intensity ($d_{C}=28$ nm, $\sigma_{d_{C}}=0.3$, $K_{eff}=-15$ kJ/m^3^).

The impact of varying $K_{eff}$ is stronger for large-sized MNP. Specifically, the signal intensity is up to ~50% larger for $d_{C}=28$ nm compared to $d_{C}=20$ nm (compare to Figure 3a–d). When varying $K_{eff}$ values between $\left(-3,\;\ldots ,\;-25\right)$ kJ/m^3^, we found for the strongest impact at $d_{C}=28$ nm with a maximum decrease in signal intensity of up to 20% and 25% for $\sigma_{d_{C}}=0.05$ and $\sigma_{d_{C}}=0.3$, respectively (Figure 3b,d). To further analyze the competition between $d_{C}$ and $K_{eff}$ in FMMD signal generation, we compare the maximum (peak) signal intensity values of the first intermodulation signals ($f_{1}+f_{2}$) for each $K_{eff}$-value that was simulated (extracted from Figure 3) for two different core sizes ($d_{C}=20$ nm & 28 nm) and size distributions ($\sigma_{d_{C}}=$0.05 & 0.3), see Figure 7. From direct comparison of $d_{C}=20$ nm vs. $d_{C}=28$ nm (Figure 7a), one sees the larger particles at approx. (80–100)% while the mid-size particles are almost constant around approx. 50%. This demonstrates that the size-dependency of FMMD signal generation is clearly dominating the effect of $K_{eff}$. Furthermore, the strongest signal for $d_{C}=28$ nm is observed for $K_{eff}^{max}=-15$ kJ/m^3^, similarly for both size distribution widths (Figure 7b, peaking at 100% for $\sigma_{d_{C}}=0.3$ and 98.3% for $\sigma_{d_{C}}=0.05$). Also, at the extremal values in the variation range ($K_{eff}<-5$ kJ/m^3^ and $K_{eff}>-20$ kJ/m^3^), a decrease by >10% is found, as already observed in Section 3.1. For $d_{C}=20$ nm, the maximum signal intensity is generally less dependent on $K_{eff}$, but nevertheless does peak at $K_{eff}^{max}=-21$ kJ/m^3^ in case of both size distribution widths (Figure 7c, peaking at 54.0% for $\sigma_{d_{C}}=0.3$ and 49.4% for $\sigma_{d_{C}}=0.05$).

Both effects (peak value and drop at extremal values) are slightly more pronounced for larger size distribution widths, which is attributed to the dominating contribution from larger-than-mean particles, as discussed in the beginning of this section.

It is insightful to further understand the size-dependent nature of the magnetic anisotropy under a fundamental framework of anisotropy contributions: The magnetic anisotropy of a solid may comprise up to four contributions: (bulk) magneto-crystalline anisotropy (from periodic order in crystal lattice and spin-orbit interactions), $K_{B}$, shape anisotropy (from stray-field interactions at the surface of differently shaped bodies), $K_{sh}$, stress anisotropy (from mechanical stress on crystal lattice), $K_{st}$, and surface anisotropy (for nano-sized magnetic objects with a relatively large surface to volume ratio), $K_{S}$ [54,55]. While $K_{sh}$ and $K_{st}$ are usually negligible, surface anisotropy $K_{S}$ can noticeably enhance magneto-crystalline bulk anisotropy, $K_{B}$, for MNP in the range of $d_{C}\sim 10$ nm [56]. Then, the effective anisotropy becomes core-size-dependent and can be described in first approximation for spherical MNP as [57]:

(7) $$K_{eff}\left(d_{C}\right)=K_{B}+\frac{6}{d_{C}}\cdot K_{S}$$

Here, both $K_{B}$ and $K_{S}$ are expected to be less than zero for magnetite. Equation (7) has two important implications: (I) that (the amount of) effective anisotropy increases for smaller core size particles, and (II) that nanostructured objects (for which Equation (7) holds) always have larger-than-bulk effective anisotropy values.

Assuming that maximum signal intensity is a key design criterion for MNP properties, both assumptions can be applied to our study (compare to Figure 7): For both particle core sizes at which $K_{eff}$ was varied ($d_{C}=20$ nm & $d_{C}=28$ nm), the optimal values read $|K_{eff,20nm}^{max}|=|-21$ kJ/m^3^$|>|K_{eff,28nm}^{max}|=|-15$ kJ/m^3^$|>K_{eff,{Fe}_{3}O_{4}}^{bulk}=-11$ kJ/m^3^, complying with implications (I) and (II). This agreement with Equation (7) confirms the $d_{C}$-dependency of the $K_{eff}$-dependency in FMMD signal generation and shall be considered in future investigations (see Section 4.3).

### 4.2. FMMD Dependency on External Applied Field Parameters: $H_{2},f_{1}$

Besides optimizing the design rules of MNP applied for FMMD (as discussed in Section 4.1), which are generally restricted by the practical limitations of particle synthesis, the subsequent optimization step is the choice of excitation field parameters for these particles as discussed here. The effect of drive field amplitude ($H_{2}$) is also important for the FMMD signal generation, since it governs the evolution of the characteristic shape of the intermodulation signals profiles ($f_{1}+nf_{2}$) (compare to Section 3.2, especially Figure 5).

For even mixing harmonics ($f_{1}+nf_{2},\;n=2,\;4,\ldots$), the optimization of the excitation field is simple since the maximum nonlinear response is obtained at zero offset field, *H*_0_ = 0. The response signal increases with increasing drive field amplitude *H*_2_ until it starts to saturate when it reaches the characteristic field [55]:

(8) $$\mu_{0}H=k_{B}\cdot \frac{T}{m_{p}},$$

where *T* is the absolute Temperature, *k_B_* is Boltzmann’s constant and $m_{p}=M_{s}d_{c}^{3}/6$ is the saturation magnetic moment of a particle with core diameter *d_c_* and saturation magnetization *M_s_*. For odd mixing harmonics ($f_{1}+nf_{2},\;n=1,\;3,\;\ldots$), it is more complicated because the optimum offset field *H*_0_ increases with increasing drive amplitude *H*_2_, as depicted in Figure 5.

**Figure 8:**
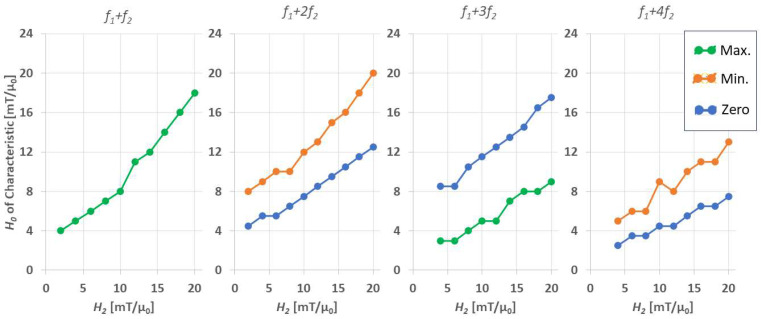
Characteristics extracted from the FMMD signal intensity profiles given in Figure 5, namely the maximum (Max., green) minimum (Min., orange) and zero-crossing (Zero, blue) for the first mixing frequencies $f_{1}+nf_{2};\;n=1,\;2,\;3,\;4$. For higher mixing frequencies (n=3, 4) the characteristics are indiscernible from Figure 5 for $H_{2}=2$ mT/µ_0_.

To elaborate the influence of $H_{2}$ further, we extract the offset field values, $H_{0}$, for which the FMMD signal profiles show the following characteristics depending on the mixing harmonics from Figure 5: the maximum (peak) intensity for $f_{1}+f_{2}$, the minimum and zero-crossing for $f_{1}+2f_{2}$, the maximum and zero-crossing for $f_{1}+{3f}_{2}$ and the minimum and zero-crossing for $f_{1}+{4f}_{2}$, summarized in Figure 8. As shown there, the offset field values for both FMMD intensity profile maximum and minimum increase with increasing the drive field $H_{2}$. The same trend is observed for the zero crossing offset field amplitude.

A possible explanation could be that the stronger the drive field, the more small particles contribute to the signal. As the characteristic points of the nonlinear magnetic response regime of the small particles lie at larger fields, the maximum is shifted in that direction, when reaching the characteristic field according to Equation (8). In addition, the optimum excitation field vector (combining *H*_0_, *H*_2_) is expected to also depend on the parameters of the lognormal core-size distribution, i.e., median core diameter *d*_0_ and distribution width *σ*, lognormal as described in Section 4.1 above. In case of median particle diameter *d*_C_ = 20 nm and narrow distribution width *σ* = 0.05, the amplitude *H*_2_ of the low-frequency drive field can be chosen according to the calculated optimum value depicted in Figure 5. Larger drive field amplitudes *H*_2_ require larger offset fields *H*_0_, but similar to the case of even harmonics, saturation is reached when the drive field approaches the characteristic field. In combination with the result of Section 4.1 that large particles dominate the FMMD signal, choosing large(r) particles of narrow size distribution can also optimize the FMMD setup requirements, as lower fields are needed to generate a contribution to the overall signal.

The fact that the FMMD signal shows negligible excitation frequency ($f_{1}$) dependence is attributed to the fact that for the given parameters (see Table 1), the resonance frequencies of both Néel ($f_{N}\sim (100-1000$) kHz) and Brownian relaxation (below 1 kHz) are well above or below *f*_1_ [40]. Therefore, the field-dependence of Néel [58] and Brownian [59] relaxation influences the FMMD signal only weakly, as shown here for commonly used FMMD field settings. This might change, if FMMD is applied for theranostics by combination with particle heating that requires higher frequencies of several hundred kilohertz, for example, for application, the feasibility of which was recently demonstrated for MPI–MFH combination [60,61]. This, however, is outside the scope of this paper.

### 4.3. Limitations and Potential Improvements of the Simulation Framework

The present study identifies key input parameters contributing to FMMD signal generation from isolated parameter variation; however, the following limitations are faced, which we will directly turn into potential improvements for future investigations:

(1) As identified in Section 4.1 (discussing the $d_{H}$-dependence), the present simulation framework does not consider particle agglomeration/clustering. However, it is becoming more and more evident that agglomerations (or clusters) play a significant role in MNP systems, either globally (non-directional) [62,63] or as a precondition by purposeful alignment of MNP [64,65]. Recently, it has been demonstrated that agglomeration in a similar simulation framework can be included [66]. However, integration of agglomeration of MNP is indisputably linked to the consideration of magnetic dipole–dipole interactions [67,68], as well as a (more) complex description of the hydrodynamic size [52,69]. Even though the present framework is capable of including magnetic dipole–dipole interactions [27,31], it is not yet sufficiently optimized to be run time-efficiently, since the incorporation of such interactions increases computation time exponentially [34].
(2) As identified in Section 4.2, the variation of core size, $d_{C}$, cannot be separated apart from that of effective anisotropy, $K_{eff}$. Therefore, future investigations shall incorporate Equation (7) in the simulation framework to investigate the core-size dependency of the anisotropy constant further.

## 5. Conclusions

In the present study, we studied the dependency of FMMD signal generation via coupled Néel–Brownian dynamic relaxation simulations. We separately varied the four intrinsic particle properties, core size ($d_{C}$), effective anisotropy ($K_{eff}$), size distribution width (${\sigma_{d}}_{C}$) and hydrodynamic size ($d_{H}$), as well as the external field parameters of excitation frequency ($f_{1}$) and drive field amplitude ($H_{2}$). In summary, we found the following:

(1) Core-size effects are strongly dominating the FMMD signal generation, above all other analyzed intrinsic particle properties. This is visible both directly in a steady increase in FMMD signal intensity with increasing $d_{C}$, as well as indirectly by increasing the core-size distribution and thereby introducing dominating contributions from few large particles.
(2) The effective anisotropy does have a remarkable effect of FMMD signal generation, but is secondary to that of (larger) core sizes. However, there is evidence that the effective anisotropy itself is core-size-dependent, such that $K_{eff}$ is increasing for smaller sized particles, as summarized in Figure 7.
(3) The drive field amplitude is dominating the shape of the FMMD signal profile. For given magnetic particle ensembles, in case of even mixing terms $f_{1}+2f_{2}$, $f_{1}+4f_{2}$, …, the offset field should be zero, and the drive field amplitude should be turned up to the characteristic field of the ensemble. In case of odd terms $f_{1}+f_{2}$, $f_{1}+3f_{2}$, …, the combination of drive field amplitude and static offset field value needs to be optimized, as summarized in Figure 8.
(4) The hydrodynamic size, as well as the excitation frequency, does not show any noticeable effect on FMMD signal generation.

The implications from our simulative study can be conveniently used in MNP design for FMMD tracers, as well as for FMMD setup design whenever optimal signal generation abilities are desired. Combining findings (1) and (3) from the above strongly suggests choosing large(r) particles of $d_{C}>25$ nm with narrow size distributions (σ<0.1) to attain optimal signal intensities at comparatively low drive fields. Future studies with this simulation framework will focus on combining the yet isolated parameter variation (as performed here) to an ideally unified description of MNP properties. Concretely, this means integrating core-size-dependent effective anisotropy and magnetic dipole–sdipole interactions.

## Figures and Tables

**Table 1:** Key parameter settings and key parameters varied (bold face) for each simulation run.

Parameter	$d_{C}$ [nm]	$\sigma_{d_{C}}$ [--]	$d_{H}$ [nm]	$K_{eff}$ [kJ/m^3^]	$H_{2}$ [mT/µ_0_]	$f_{1}$ [kHz]
Core size $d_{C}$ [nm]	**10, 12, …, 30**	0.05	130	−11	16	40
Core-size distribution width $\sigma_{d_{C}}$ [--]	20	**0.05, 0.1, …, 0.5**	130	−11	16	40
Anisotropy constant $K_{eff}$ [kJ/m^3^]	**20, 28**	**0.05, 0.3**	130	**−3, −5, … −25**	16	40
Hydrodynamic size $d_{H}$ [nm]	20	0.05	**20, 35, …, 200**	−11	16	40
Drive field amplitude $H_{2}$ [mT/µ_0_]	20	0.05	130	−11	**2, 4, …, 20**	40
Excitation frequency $f_{1}$ [kHz]	20	0.05	130	−11	16	**16, 18, …, 48**

Other parameters (fixed): Excitation field amplitude $H_{1}=1.2$ mT/µ_0_; drive frequency $f_{2}=2$ kHz; saturation magnetization (bulk Fe_3_O_4_) $M_{S}=476$ kA/m; viscosity of the water η=0.89 mPa·s, operating temperature T=310 K.

**Table 2:** Qualitative comparison of the dependency on FMMD signal generation on the key parameter varied. The (suspected) dominating effect across all six key parameters is marked in bold (see Discussion Section 4 for details).

Parameter ↓ | Effect →	Peak Intensity	Peak Width	Shape of Profile
Core size $d_{C}$ [nm]	**Strong**	**Strong**	Moderate
Core-size distribution width $\sigma_{d_{C}}$ [--]	Weak	Moderate	None
Anisotropy constant $K_{eff}$ [kJ/m^3^]	Moderate	None	None
Hydrodynamic size $d_{H}$ [nm]	None	None	None
Drive field amplitude $H_{2}$ [mT/µ_0_]	Moderate	None	**Strong**
Excitation frequency $f_{1}$ [kHz]	None	None	None

## Data Availability

Source code used for general particle relaxation dynamics simulations can be accessed from https://github.com/cshasha/nano-simulate (accessed on 28 January 2024).
